# Biological and Biochemical Basis of the Differential Efficacy of First and Second Generation Somatostatin Receptor Ligands in Neuroendocrine Neoplasms

**DOI:** 10.3390/ijms20163940

**Published:** 2019-08-13

**Authors:** Federico Gatto, Federica Barbieri, Marica Arvigo, Stefano Thellung, Jessica Amarù, Manuela Albertelli, Diego Ferone, Tullio Florio

**Affiliations:** 1 Dipartimento di Medicina Interna & Centro di Eccellenza per la Ricerca Biomedica (CEBR), Università di Genova, 16132 Genova, Italy; 2 Istituto di Ricerca e Cura a Carattere Scientifico (IRCCS) Ospedale Policlinico San Martino, 16132 Genova, Italy

**Keywords:** somatostatin receptors, somatostatin receptor ligands, pituitary adenomas, neuroendocrine tumors

## Abstract

Endogenous somatostatin shows anti-secretory effects in both physiological and pathological settings, as well as inhibitory activity on cell growth. Since somatostatin is not suitable for clinical practice, researchers developed synthetic somatostatin receptor ligands (SRLs) to overcome this limitation. Currently, SRLs represent pivotal tools in the treatment algorithm of neuroendocrine tumors (NETs). Octreotide and lanreotide are the first-generation SRLs developed and show a preferential binding affinity to somatostatin receptor (SST) subtype 2, while pasireotide, which is a second-generation SRL, has high affinity for multiple SSTs (SST_5_ > SST_2_ > SST_3_ > SST_1_). A number of studies demonstrated that first-generation and second-generation SRLs show distinct functional properties, besides the mere receptor affinity. Therefore, the aim of the present review is to critically review the current evidence on the biological effects of SRLs in pituitary adenomas and neuroendocrine tumors, by mainly focusing on the differences between first-generation and second-generation ligands.

## 1. Introduction

Somatostatin (somatotropin release–inhibiting factor, SRIF) is an endogenous, and ubiquitous hormonal peptide [1] acting though a family of five G-protein-coupled receptors, named somatostatin receptors (SST) 1 to 5, and represents one of the main physiological inhibitors of endocrine and exocrine hormone secretion [2]. SRIF rapidly showed a widespread physiological activity related to the diffuse expression of its receptors throughout the body [3,4]. In particular, SRIF is able to modulate immune responses [5,6] and gastro-enteric-pancreatic cell activity [7]. It also shows neurotransmission and neuromodulatory functions. Particularly, SRIF is mainly involved in learning and memory processes [8,9,10], related to β-amyloid clearing [11,12], and in chronic pain modulation [13]. However, while SRIF itself exhibits effects on neurotransmission, evidence for endogenous SRIF release from SRIF-containing interneurons is lacking [14]. In the central nervous system (CNS), the characterization of SST signaling is also complicated by the identification of a novel peptide, cortistatin, able to activate all the five SSTs [15]. Subsequently, SRIF was recognized to reduce cell proliferation, increase apoptosis, and inhibit angiogenesis in most tumor tissues [2,3,4,16]. Thus, the interest in the potential use of SRIF as an anti-proliferative agent grew in recent years. This novel potential pharmacological use, despite being preclinically tested with unique results in several human solid tumors, including breast and lung carcinomas and gliomas [3,4], acquired particular interest for neuroendocrine tumors, including both pituitary adenomas and gastro-entero-pancreatic (GEP), as well as thoracic neuroendocrine neoplasms (NENs). However, SRIF is not a useful tool in clinical practice due to both its short circulating half-life (<3 min in human serum), resulting in the need for continuous parenteral administration, and to the post-infusion rebound observed for a number of target hormones, such as growth hormone (GH) and insulin [17,18].

Therefore, synthetic somatostatin receptor ligands (SRLs) have been designed based on the primary SRIF structure, in order to overcome the above-mentioned drawbacks. To date, three SRLs have been already approved in clinical practice due to anti-secretory activity in hormone-secreting pituitary adenomas and NENs [19,20,21,22]. In particular, the SRIF analogues octreotide (OCT) and lanreotide (LAN) have been approved by the US Food and Drug Administration (FDA) and the European Medicines Agency (EMA) for treating patients with advanced GEP NENs, after two phase 3 clinical trials [23,24] showed a significant increase in the progression-free survival (PFS) of treated patients. On the other hand, pasireotide (PAS), which is a second generation SRL approved for treatment of GH-secreting and adrenonocorticotropic hormone (ACTH)-secreting pituitary adenomas, is still under evaluation for NENs.

NEN patients demonstrated a significant benefit from the use of OCT and LAN, which resulted in a prolonged disease control associated with a satisfactory safety profile.

However, the wide range of cell types expressing SSTs, including tumors of non-endocrine origin, and the observation that SRLs display anti-proliferative activity in preclinical models of these tumors [3,25,26,27,28], open the possibility of a wider anti-tumor use for these compounds including in patients suffering from non-endocrine neoplasia. However, no successful trials have been reported with the use of SRLs as anti-proliferative agents in non-endocrine tumors’ patients so far.

Notwithstanding the negative results reported for non-endocrine tumors, several studies thoroughly characterized the expression pattern of the five SST subtypes in human tumors and the molecular intracellular mechanisms by which each of these receptors activates anti-secretory and anti-proliferative signals in tumor cells.

### 1.1. Somatostatin Receptor Signaling

**Figure 1:**
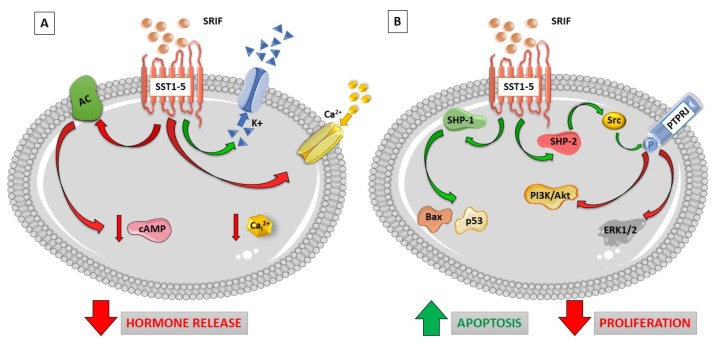
Diagram of the main intracellular signaling pathways triggered by agonist binding to somatostatin (SRIF) receptors to exert anti-secretory and anti-proliferative effects. (**A**) Anti-secretory activity of SRIF is regulated by: i) the inhibition of adenylyl cyclase (AC), lowering cyclic adenosine monophosphate (cAMP) levels; ii) the inhibition of voltage-dependent Ca^2+^ channels; and iii) the activation of outward K^+^ channels, leading to cell membrane hyperpolarization. (**B**) Anti-proliferative effects of SRIF are mediated by the activation of the protein tyrosine phosphatases Src homology 2 domain-containing protein tyrosine phosphatase 1 and 2 (SHP-1 and SHP-2) and the protein tyrosine phosphatase receptor type J (PTPRJ). SHP-1 triggers intracellular pro-apoptotic signals involving the induction of p53 and Bax, while SHP-2 activates the tyrosine kinase Src that induces the phosphorylation of PTP*η*, which, in turn, dephosphorylates PI3K/Akt and ERK1/2, impairing cell proliferation. Green arrows: Activated pathway. Red arrows: Inhibited pathways.

Soon after the molecular cloning of a family of five SSTs with a specific, although sometimes overlapping, expression pattern, most studies focused on the identification of specific intracellular signaling modulated by each receptor subtype. However, after more than 20 years from the first receptor cloning, as far as most of the second messenger systems is concerned, most SST subtypes seem to activate a similar signaling cascade (Figure 1 depicts the better characterized intracellular pathways modulated by SSTs). In particular, all members of this receptor family are G-protein coupled receptors (GPCRs) acting through inhibitory G proteins (Gi/Go) [29]. Belonging to the inhibitory receptors, SSTs are able to inhibit cAMP production and PKA activation [30,31,32], which is associated with reduced activity of voltage-dependent Ca^++^ channels. The latter activity was reported in different cell systems to be direct, mediated by the α subunit of Gi/Go [33], or induced indirectly either by the reduced PKA activity or by the βγ subunit-dependent activation of inward-rectifier K^+^ channels [4,34]. The net result of these combinations of intracellular signals is plasma membrane hyperpolarization, which leads to all the anti-secretory activity of SRIF, not only in endocrine cells but also in neurons [35]. This contributes to the cognitive effects of this peptide in the CNS [8]. The same mechanisms are currently considered the determinants of the anti-secretory activity of SRLs in hormone-secreting tumor cells, including pituitary adenomas and NENs.

On the other hand, several intracellular pathways common to most SST subtypes were identified with regard to their anti-proliferative effects. In particular, the activation of these receptors mainly exerts cytostatic effects due to the up-regulation of cyclin-dependent kinase (CDK) inhibitors (either p21^cip1/Waf1^ or p27^kip2^, according to the cell types analyzed) [36,37,38,39] or the zinc finger protein (Zac1) [40]. Of note, the observed up-regulation of both CDK inhibitors and Zac1 depend on the modulation of MAP kinase activity, which is one of the main pathways controlled by tyrosine kinase receptors to induce cell proliferation [39,41,42,43,44,45].

However, distinct mechanisms were observed after SST activation in different cell systems, which causes either inhibition or activation of MAP kinases. In most studies, an inhibitory effect was observed. In fact, the activation of all five SSTs leads to protein tyrosine phosphatase (PTP) activation, as initially discovered by Pan & Coll. [46], to inhibit tyrosine kinase-related mitogenic signaling pathways. Some PTPs were then associated with specific SST activity by biochemical assays, including the cytosolic Src homology region 2 domain-containing phosphatase 1 (SHP1) and 2 (SHP2), and the human receptor-like tyrosine phosphatase PTPRJ (or, as shown in most of the studies, its murine homologue PTPη) [47,48]. In primary cultures of GH-secreting and non-functioning pituitary adenomas, LAN induces anti-proliferative activity through the induction of a PTP activity [49,50], while OCT triggers SHP1 activity in GH4C1 rat pituitary adenoma cell line [51]. SHP1 and/or SHP2 activities are also induced by SST_1_ [52,53], SST_2_ [54,55], SST_3_ [55], and SST_4_ [55,56], while PTPη is activated by SST_1_, SST_2_, and SST_5_, as shown in cells endogenously expressing the different SST subtypes [41]. The molecular mechanisms connecting SSTs and PTPs have not been completely clarified, but likely are not directly induced by G protein activation and involve the modulation of different intracellular transducers [47]. However, one of the main targets of the SRIF-induced PTP activity are the MAP kinases ERK1/2, whose dephosphorylation leads to an inhibition of growth factor activity and cell proliferation arrest [41,42,44,57,58]. Other studies reported that all SSTs can activate phospholipase C (PLC) [59], which promotes inositol-1,4,5-trisphosphate formation and Ca^++^ release from intracellular stores, and activates protein kinase C (PKC). This signaling causes the activation of MAPK pathway [60], although SST_1_ and SST_5_-dependent anti-proliferative effects are observed in this situation due to the ability of ERK1/2 to induce up-regulation of p21^cip1/Waf1^ [37]. It has to be remarked, however, that most of the studies showing an SRIF-dependent activation of the PLC/ERK1/2 pathway were performed in heterologous SST-expressing cells. Therefore, the relevance of this evidence in human cells in vivo is still unclear.

Lastly, SST_2_ and SST_4_, but not SST_3_, were shown to increase the activity of p38, which is another MAP kinase family component mainly endowed with anti-proliferative and pro-apoptotic effects that causes the overexpression of p21^cip1/Waf1^ and growth arrest [61].

Heterologous SSTs expression was also reported to induce pro-apoptotic effects [62]. In particular, this was shown for SST_3_, which, when expressed in CHO-K1 cells, causes the activation of the apoptosis-related proteins p53 and Bax [63]. Similarly, besides cytostatic effects, SST_2_ induces the activation of pro-apoptotic signaling, through the inhibition of the anti-apoptotic factor Bcl-2 [64]. The signaling differences observed in different cell types, and, in particular, the different signaling observed after SSTs transfection, support the notion that, besides the specific biochemical features of receptors, the responses that can be obtained are also extremely dependent on the cell context where the receptors are activated.

Another relevant pathway, related to SRIF anti-tumor activity, is its ability to directly inhibit neoangiogenesis in different tumors [4], including those of a neuroendocrine origin [65].

This is mainly a direct effect on vessel formation, as demonstrated in in vivo studies [66], and involved the activity of SST_1_ [67], SST_2_ [68], and SST_3_ [69]. The specificity of the SST-dependent anti-angiogenic effect, when compared to the direct anti-tumor activity was demonstrated, by the observation that it was, in most cases, independent of PTPs, but it involves the inhibition of cAMP accumulation [70] and, more importantly, the activity of endothelial nitric oxide synthase (eNOS) and the consequent generation of nitric oxide (NO) [69]. Importantly, the activation of all SSTs, except for SST_4_, was shown to be able to inhibit the eNOS-dependent or neural NOS-dependent NO production, independently of the mechanisms by which the enzyme was induced, which suggests a direct modulation of their activity [71,72]. Conversely, truncated isoforms of SST_5_ (SST_5_TMD4), recently identified in different tumor histotypes as possible inhibitors of canonical SRIF activity on its receptors [73], were reported to stimulate the pro-angiogenic pathways. This results in an increased lymphatic metastasis in breast cancer [74].

Lastly, it was reported that SST_1_, SST_3_, and SST_4_ activation leads to the inhibition of the Na^+^/H^+^ exchanger (NHE1), causing intracellular acidification [75], which may be responsible for anti-migration activity observed by SST activation in several tumor cells [45]. In agreement with this observation, SST_1_ activation was also reported to inhibit Rho GTPase, involved in the cytoskeleton reorganization, cell adhesion, and cell motility, and regulated by NHE1 activity [76].

### 1.2. Somatostatin Receptor Homo-Dimerization and Hetero-Dimerization

Dimerization is a novel frontier in the regulation of the activity of GPCRs. In fact, this receptor family activity is modulated by homo-dimerization or hetero-dimerization, the latter possibly occurring between two GPCRs of the same or different families. While a variable degree of dimerization may occur according to the GPCR family involved, this process can represent either a constitutive event, occurring in the endoplasmic reticulum during receptor synthesis and representing a prerequisite for their correct membrane insertion, or, in other situations, dimerization can be induced by ligand binding [77]. It is currently accepted that heterodimerization results in the diversification of GPCR functioning, the modification of ligand binding affinity, intracellular signaling, receptor internalization, desensitization, and recycling [78].

After heterologous expression in both CHO-K1 and HEK-293T cells, SST_2_ and SST_3_ form constitutive homodimers, which progressively dissociate in the presence of increasing concentrations of SRIF, independently form the receptor concentration [79,80]. Conversely, in the same cell lines, human SST_5_ does not form constitutive dimers, which are formed only after treatment with SRIF [81]. A different response was observed for SST_1_, which never forms homodimers [82].

More complex results were provided with regard to heterodimerization. Several pharmacological evaluations showed different responses to SST agonists according to the receptor repertoires in a given cell, which is possibly dependent on the interaction between different receptor subtypes, although not formally demonstrated. For example, in C6 glioma cells, natively expressing all SSTs but SST_4_, all endowed with anti-proliferative activity via the activation of PTPη and the inhibition of ERK1/2, the concomitant activation of SST_1_ and SST_2_ caused a synergistic cytostatic effect representing a possible heterodimerization effect. This did not occur with SST_2_ and SST_5_, whose combined activation resulted in a response resembling the SST_5_ individual effects [41].

More direct evaluations were also performed after cell transfection with individual receptors. It was observed that complexes formed among SST subtypes are formed following specific structural determinants, rather than occurring in the presence of random combinations. Thus, it was proposed that these events are highly selective and not all the receptor dimer combinations are possible. The first heterodimers observed are the complexes between SST_2_ and SST_3_ [80], and SST_1_ and SST_5_ [83]. Immunoprecipitation studies in prostate carcinoma cell lines showed that SST_1_/SST_2_ and SST_2_/SST_5_ heterodimers can be generated by natively expressed molecules. In these cells, receptor complexes are constitutively present within plasma membrane and their formation rate is increased by bispecific agonist binding (BIM-23704 and BIM-2324, respectively), which potentiates anti-proliferative responses [84]. In some cases, SST heterodimerization subverts receptor functioning, since SST_2_/SST_3_ heterodimer formation causes loss of SST_3_ activity while SST_2_ function is not changed [80]. On the other hand, SST_1_/SST_5_ dimers show higher affinity for SRIF as compared to individual receptors [83]. SST_3_ within dimers loses its internalization ability, conferring to SST_2_/SST_3_ heterodimers that increased resistance to agonist-dependent desensitization [80]. More recently, co-immunoprecipitation studies performed in MDA-MB-435S breast cancer cells, reported the formation of SST_1_/SST_4_ heterodimers in basal condition, which was increased after treatment with SRLs. This resulted in a potentiation of the anti-proliferative activity [85].

SST_2_/SST_5_ heterodimers are particularly relevant since these receptors are the target of the clinically approved SRLs. In co-transfected HEK-293 cells, selective activation of SST_2_, but not that of SST_5_ or the coactivation of both SST subtypes, was able to favor the association between these SST subtypes. However, SST_2_ activation caused a higher inhibition of cAMP and a more potent modulation of ERK1/2 and p27^Kip1^, which resulted in enhanced inhibition of cell proliferation, in the presence of both receptors than in cells expressing SST_2_ alone [86]. Moreover, upon agonist-dependent activation, monomeric SST_2_ rapidly desensitize due to β-arrestin binding. Heterodimers display destabilization of the β-arrestin-receptor interaction, which increases the rate of the membrane recycling of internalized receptors [86]. This mechanism was proposed as one of the molecular determinants for SRL effectiveness in controlling pituitary tumors and the absence of tolerance seen in patients undergoing long-term SRL administration.

SST functioning is also modified by dimer generation with components of other GPCR families, including opioid [87] and dopamine receptors [88,89]. In particular, SST_2_ is able to heterodimerize with the μ opioid receptor-1 (MOR1), after heterologous transfection in HEK-293 cells [87]. This event does not change the signaling properties of the receptors but causes the desensitization and endocytosis of both the components of the dimer in response to SRLs [87]. SST_5_ and dopamine-2 receptor (D2R) interaction was demonstrated in co-transfected CHO-K1 cells. In the absence of ligand stimulation, these receptors do not heterodimerize. However, in the presence of either SST-specific or D2R-specific ligands, a significant association between the two receptor subtypes was observed [88,89]. This heterodimer displays a completely different pharmacology as compared to individual receptors, with increased (or decreased) affinity to SRIF in the presence of D2R agonists (or antagonists) able to potentiate (or inhibit) the ability of SRLs to affect cAMP production [88]. More recently, heterodimerization between SST_5_ and β1 adrenergic receptor (β1-AR), was also shown in co-transfected HEK-293 cells. SST_5_/β1AR heterodimers, already detected in untreated conditions, were highly increased in the presence of agonists for both receptors. However, the individual binding of β1-AR or SST_5_ caused dimer dissociation. After co-treatment, β1-AR increase in cAMP production was the predominant effect, while ERK1/2 activity was predominantly regulated by SST_5_ [90].

Thus, heterodimerization among SSTs, or with other GPCRs, can represent a significant interfering factor to be taken into account when patients are treated with ligands able to bind more receptors, and it could explain the different tissue responses (including those of tumor origin) characterized by specific receptors repertoire. Since the clinically approved SRLs (OCT, LAN, and PAS, see next paragraph) display high affinity binding to multiple SSTs, and receptor repertoire of the target cells included the presence of apparently off-target receptors able to heterodimerize with SSTs, which could dramatically change the responses to these drugs. However, this issue has not yet been deeply analyzed and will require further investigation in the coming years.

## 2. “Old” and “New” Somatostatin Receptor Ligands

**Figure 2:**
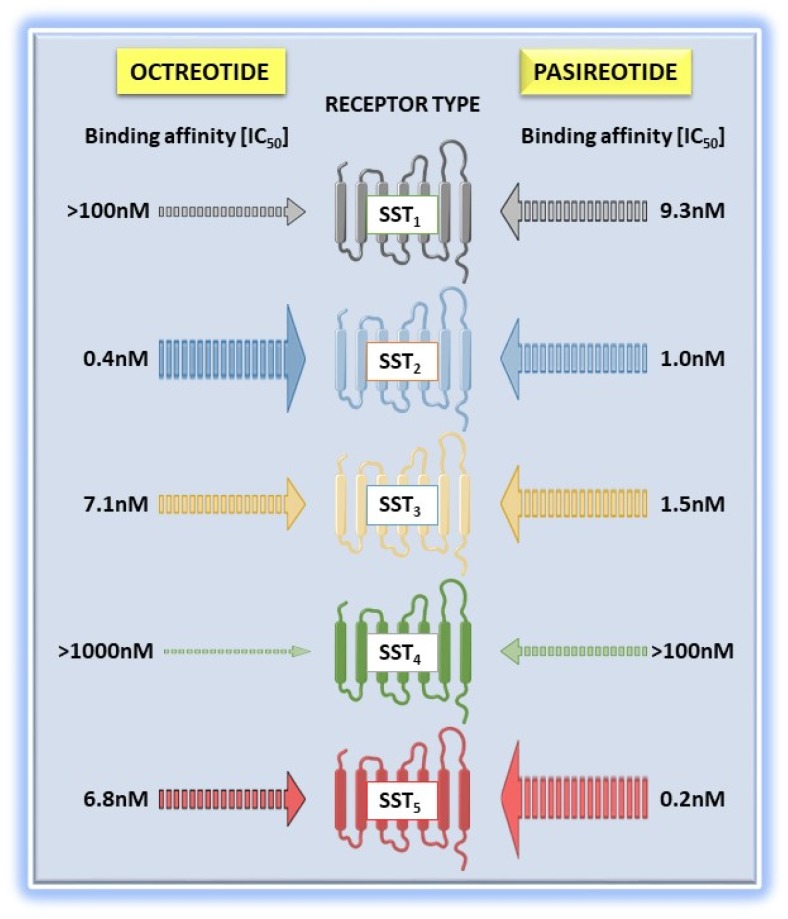
Octreotide and pasireotide binding affinity toward human somatostatin receptor subtypes.

As mentioned in the previous paragraphs, anti-secretory and anti-proliferative effects of native SRIF are currently exploited using synthetic SRLs, among which OCT and LAN were the first drugs clinically approved [18,91]. They are both small molecules (octapeptides), which retain the Cys–Cys bridge present in native SRIF and then stabilizing the structure substituting a Tryptophan (Trp) with its D-enantiomer (D-Trp). Therefore, these compounds show enhanced half-life compared to SRIF (about 2 h for OCT and 90 minutes for LAN) as well as lower clearance, which results in longer duration of action and long-lasting biological activity [18,92]. Both compounds show a preferential binding affinity to SST_2_. As for the other SST subtypes, OCT has moderate affinity for SST_5_ and a weak interaction with SST_3_, while LAN shows a slightly more pronounced affinity to SST_5_. Of note, differently from native SRIF, both octapeptides have a negligible binding to SST_1_ and SST_4_ (Figure 2) [93,94].

OCT and LAN became clinically available in the last 30 years, with different formulation developed, tested, and then approved by both EMA and FDA [95]. First, in 1988, a short-acting formulation of OCT, administered subcutaneously (s.c.) or intravenously (i.v.), received approval for treating acromegaly patients in Europe, while the long-acting repeatable formulation (LAR, long-acting repeatable) was introduced and approved in 1995. The technology underlying OCT LAR formulation consists in a depot preparation in which OCT molecules are encapsulated in microspheres of a biodegradable polymer. The LAR formulation allows clinicians to treat patients with an intramuscular (i.m.) injection every four weeks, instead of the standard three s.c. administrations/day required for the short-acting drug. A single OCT LAR injection results in an initial peak within 1 hour of administration, with a progressive decrease in the following 12 h, and a subsequent second-release phase showing a sustained drug release reaching a plateau between days 14-42 [96]. Of note, the steady-state concentrations of OCT in serum are reached after three LAR injections. Currently, OCT LAR is commercially available in three different dosages (10, 20, and 30 mg), with a maximum allowed dose of 40 mg (two 20 mg injections in acromegaly) every four weeks.

As far as LAN is concerned, the first approved formulation consisted in a sustained-release formulation (LAN SR), developed using a microparticle-based delivery system. LAN SR is available as a power for suspension for intramuscular injection at a dosage of 30 mg. Starting treatment schedule is recommended as 30 mg/14 days, but subsequent injections may be given every seven to 10 days, depending on patients’ responses. However, in 2001, another LAN formulation, LAN Autogel, has been approved by EMA for treating acromegaly patients [95]. LAN Autogel represents the first available sustained-release formulation based on self-assembling nanotube technology [97], available in prefilled syringes (dosages 60-90-120 mg) administrated by deep s.c. injection every four weeks, even though administration frequency can be modulated according to the patient’s response (i.e., every three to five weeks) [98]. Therefore, the improved patients’ compliance, determined that, currently, LAN Autogel has almost completely replaced the SR formulation in daily clinical practice. LAN Autogel has a different release pattern than OCT LAR, since peptide monomers are slowly released by the nanotubes after injection. In more detail, the drug pharmacokinetic is characterized by an initial acute increase of serum LAN concentrations, which reaches a peak during day 1, followed by a gradual decrease during the following four weeks [99].

Lastly, PAS represents a second-generation SRL. The molecule consists of a stable cyclohexapeptide with a long half-life (about 24 h), synthetized based on the SRIF structure and showing high affinity for multiple SSTs (SST_5_ > SST_2_ > SST_3_ > SST_1_). In detail, different from the first-generation SRLs, OCT, and LAN, PAS shows a binding affinity in the low nanomolar range for SST_5_ (IC_50_: 0.2 nM), SST_2_ (IC_50_: 1 nM), and SST_3_ (IC_50_: 1.5 nM) (Figure 2) [100].

Since first-generation SRLs mainly target SST_2_, but different SST subtypes are heterogeneously expressed in pituitary and neuroendocrine tumors, researchers aimed to generate a compound with a more universal binding profile for SSTs, similar to that of native SRIF. Among a number of novel compounds tested in vitro and described in the recent literature [28,101], PAS is the only SST pan-ligand that has been approved by EMA and FDA for clinical use.

Currently, two formulations of PAS are available for clinical practice: a short-acting s.c. formulation approved for treating Cushing’s disease, and a long-acting formulation (PAS LAR) for intramuscular injection developed using the same technology than OCT LAR, which was approved for treating Cushing’s disease and acromegaly [95].

Short-acting PAS is available as a solution for s.c. injection in three dosages (0.3 mg, 0.6 mg, and 0.9 mg), with a recommended starting treatment schedule of 0.6 mg twice a day. On the other hand, PAS LAR is available as powder and solvent for deep intramuscular injection at different dosages (20 mg, 40 mg, and 60 mg), with a recommended starting dose of 40 mg/four weeks and a maximum allowed dose of 60 mg/four weeks. Studies in healthy volunteers show that PAS s.c. is rapidly absorbed, with maximum plasma concentrations reaching in <1 h [102], while, similarly to that observed for OCT LAR, PAS LAR exhibits an extended-release profile with an initial burst release, a subsequent decline of plasma concentrations, and then another increase rising to a peak over approximately one week and three weeks [103].

To summarize, PAS shows a pattern of binding affinity for SSTs similar to native SRIF compared to first-generation analogs. However, despite the initial search for a compound able to closely mimic native SRIF effects, and possibly overcoming its limitations (i.e., short half-life), a number of studies already demonstrated that PAS has different functional properties compared to both SRIF and first-generation SRLs when binding SSTs, and particularly SST_2_. These differences, going beyond the different binding properties for membrane receptors, will be described in detail in the following chapter of the review.

### Differential Functional Properties of Somatostatin Receptor Ligands

First-generation and second-generation SRLs differ in their biological properties, which causes different biological responses, including SST pathway activation and modulation of receptors’ phosphorylation, internalization, and trafficking.

As mentioned above, the first clear difference resides in a broader SST binding affinity of PAS compared to both OCT and LAN [100]. Particularly, the significantly higher binding affinity of PAS for SST_5_ results in a more potent activation of different intracellular pathways. In more detail, Lesche & Coll. demonstrated that, in HEK 293 cells stably transfected with human SST_5_, PAS was superior to OCT in decreasing intracellular cAMP levels and in the stimulation of ERK1/2 phosphorylation [104]. This evidence, derived from transfected cell models, is in line with subsequent preclinical studies carried out in corticotroph pituitary cells (mainly expressing SST_5_ among all SST subtypes), which show a greater efficacy of PAS, compared to first-generation SRLs, in the inhibition of basal and/or CRH-stimulated ACTH secretion [105,106].

However, the differences between first-generation and second-generation SRLs go far beyond the mere membrane receptor binding. The specific intracellular pathways activated by SRLs, as well as their potency, is different in the different tumor types, depending on the specific SST distribution pattern, as well as signaling elements, receptor desensitization, internalization, and cross talk [107,108].

Since SST_2_ is the SST subtype mostly expressed in the majority of pituitary adenomas and NENs, and first-generation SRLs mainly target SST_2_, most studies focused on the differential activation of this specific SST subtype when investigating the biological differences between PAS and the other SRLs.

In this context, a number of studies demonstrated that, in the same cell type, PAS may elicit differential effects compared to both OCT, native SRIF, and SST selective ligands, when targeting SST_2_, possibly due to the activation of different subsets of intracellular mediators [104,108,109,110].

This phenomenon, also named biased-agonism, depends on specific agonist-receptor interactions. In this light, Cescato & Coll. observed that, in HEK 293 cells stably transfected with rat SST_2_ and rat pancreatic AR42J cells, PAS is less potent than OCT in inhibiting intracellular cAMP production. Furthermore, differently from OCT, it antagonizes SRIF modulation of intracellular Ca^++^ concentrations ([Ca^++^]i), and behaves as partial agonists for SRIF-mediated ERK1/2 phosphorylation [111]. These data were in line with previous findings showing that, in HEK cells transfected with human SST_2_, PAS induced a less potent cAMP synthesis inhibition and a lower ERK1/2 phosphorylation compared to both OCT and native SRIF [104].

Despite SRIF, OCT, and PAS show similar binding affinity for SST_2_ (within the low nanomolar range), PAS was reported to induce an SST_2_ phosphorylation pattern and trafficking rate, that were clearly different to those caused by OCT and native SRIF [104,109,110,112]. In this context, similarly to several other GPCRs, SST_2_ undergoes agonist-induced endocytosis following agonist binding. The activated receptor is then phosphorylated by G protein coupled receptor kinases (GRKs) and, subsequently, recruited by cytoplasmic proteins, named β-arrestins, which determines uncoupling between the receptor and its related G proteins [113,114]. The receptor/β-arrestin complex is then internalized by dynamin-dependent endocytosis. In more detail, regulation of SST_2_ involves the phosphorylation of specific serine (Ser) and threonine (Thr) residues identified in the C-terminal tail of the receptor. After SRIF and/or OCT stimulation, phosphorylation of Ser341, Ser343, Ser348, Thr353, and Thr354 has been observed in rat SST_2_-transfected CHO and GH_4_C_1_ cells [115], while Ser341, Ser343, Thr353, Thr354, Thr356, and Thr359 have been identified in HEK293 cells transfected with rat or human SST_2_ [112,116]. Furthermore, an agonist-dependent SST_2_ phosphorylation of the four Thr residues has been documented in rat pituitary GH_3_ cells transfected with rat SST2, rat insulinoma β-cells (INS1 cells), and rat pancreas in vivo [110]. Of note, unlike SRIF and OCT, PAS stimulates only phosphorylation of Ser341 and Ser343 residues of human SST2, which is followed by a partial receptor internalization [104,116]. In this context, GRK2 and GRK3 have been pointed out as the receptor kinases mainly involved in SST_2_ phosphorylation of C-tail Ser and Thr residues, since GRK2/GRK3 upregulation results in an increased receptor phosphorylation both after OCT and PAS treatment [110,112].

The differential SST_2_ phosphorylation induced by PAS, as compared to SRIF and/or OCT, results in a striking different β-arrestin recruitment and receptor internalization rate. PAS causes a significantly lower internalization of SST_2_ compared to both OCT and SRIF in HEK cells transfected with the human receptor, as well as in rat cell lines and endocrine tissues (rat pituitary and pancreas) [104,110,116]. Another study confirmed that, compared to SRIF and/or OCT, the degree of SST_2_ internalization following PAS application is smaller, both in terms of potency and percent maximal internalization reached after receptor activation [109] (Table 1). These data on cell lines (SST_2-_transfected or endogenously expressing the receptor) have been recently confirmed in an elegant study carried out on human pancreatic neuroendocrine tumor primary cultures [117]. The authors observed that, differently from OCT, PAS results in a rapid and transient internalization of SST_2_ followed by a persistent recycling of the receptor at the cell surface. Based on our knowledge, there are no studies published so far evaluating the role of PAS compared to OCT and/or native SRIF on SST_2_ internalization and trafficking in primary cultures of somatotroph and/or corticotroph adenomas. This could be important to further confirm the above reported findings in a reliable model of human pituitary adenomas.

However, in line with analysis of current data investigating the agonist-dependent receptor internalization, multiple studies demonstrated that, after PAS binding, less β-arrestins are recruited to the cell membrane compared to SRIF treatment, which results in a less stable complex with SST_2_ [109,110].

Since β-arrestins play a pivotal role in the desensitization-internalization processes of GPCRs, including SST_2_, the agonist-dependent recruitment of these molecules may impact receptor signaling and drug efficacy. Our group recently demonstrated that β-arrestin expression correlates with the anti-secretory efficacy of first-generation SRLs in GH-secreting adenoma, both in vitro and in vivo [118,119]. In this light, the differential properties of PAS compared to both SRIF and first-generation SRLs shed light on the importance of the agonist-induced receptor conformation in affecting receptor signaling and regulation, besides and beyond agonist-binding affinity alone.

## 3. Comparison between First-Generation and Second-Generation Somatostatin Receptor Ligands in Clinical Studies and Preclinical Models of Pituitary Tumors

### 3.1. Acromegaly

Acromegaly is a severe systemic condition mainly dependent on somatotroph pituitary adenomas. It is characterized by high plasma levels of GH and IGF-1 leading to increased mortality and morbidity [120]. Treatment of acromegaly aims to normalize GH and IGF-1 levels, which reestablishes a normal life expectancy [120]. The first line treatment in most acromegaly patients is represented by trans-sphenoid surgery since it provides instantaneous reduction of GH levels with low complication rates [121]. However, up to 50% of patients with macroadenomas do not normalize GH and/or IGF-1 levels, which, therefore, require medical adjuvant therapy [122,123].

To date, first-generation SRLs, OCT, and LAN represent the first line medical treatment of acromegaly because of the predominant expression of SST_2_ on tumor pituitary cells [124]. However, studies on long-term efficacy and safety of first generation SRLs demonstrated that only 30% to 40% of patients achieve normalization of predefined GH and IGF-1 levels during long-term treatment [125], even though a significant percentage of pituitary adenomas shows a variable degree of shrinkage during long-term SRL treatment. Moreover, only a small subgroup of GH-secreting adenomas is truly SRL resistant, mainly because SST_2_ expression is lacking or extremely low. In this context, the second-generation SRL PAS, displaying increased affinity for SSTR_5_ and other SSTRs, may have adjunctive effect over OCT or LAN.

The first in vitro study comparing the effect of OCT and PAS on GH secretion was performed on GHRH stimulated primary cultures of rat anterior pituitary cells after 3 h of treatment with the compounds [100]. This study showed that PAS-dependent GH reduction was three-fold to four-fold higher compared to both SRIF and OCT, with a half-maximal inhibitory concentration (IC_50_) in the sub-nanomolar range (0.4 ± 0.1 nM PAS vs. 1.5 ± 0.3 nM SRIF, and 1.3 ± 0.2 nM OCT). Moreover, the same study compared the efficacy of OCT and PAS in the modulation of GH, IGF-1, insulin, and glucagon levels in rat animal models, after both short-term and long-term treatment (1 hour to 126 days). OCT demonstrated to be slightly more effective than PAS in reducing GH secretion after 1 hour of treatment (median effective dose [ED_50_] 0.13 vs. 0.22 µg/kg), whereas PAS was more effective than OCT after 6 h of treatment (ED_50_ 23.7 vs. 5.5 µg/kg) demonstrating its prolonged biological action. After 126 days of continuous infusion at stable dose (10 mg/kg/h), PAS also reduced IGF-1 levels by 75% compared with placebo-treated animals, while OCT only caused a 30% decrease of IGF-1 [100].

Different results were obtained in a subsequent in vitro study evaluating the effect of PAS, SRIF, and OCT on GH secretion in primary cultures from rat pituitary cells, human fetal pituitary tissues, and human GH-secreting pituitary adenomas [126]. After treatment of murine pituitary cells, no statistical difference between OCT and PAS were observed as far as the inhibition of GH secretion (IC_50_ for PAS and OCT: 1.2 and 0.8 nM, respectively). Apparently, in line with the latter finding, results on human pituitary adenoma cell cultures (*n* = 6) demonstrated a superimposable effect of OCT and PAS in inhibiting GH secretion after 20 h of treatment. However, a careful analysis of individual human PA primary culture showed that PAS and SRIF inhibited GH secretion by more than 20% in five out of six samples, while OCT was as effective as PAS (or SRIF) only in three out of six tumors. The lack of statistical significance, as stated by authors, was likely due to the low number of samples analyzed [126]. In order to establish the prevalent SST subtype involved in PAS anti-secretory effect, the SST expression profile was evaluated in the six human pituitary cultures by reverse transcriptase (RT)-PCR. SST_2_ was expressed in all samples, while SST_5_ was expressed in only four out of six cultures. The effect of PAS in inhibiting GH secretion by more than 20%, was observed in two SST_5_-negative samples suggesting, for the first time, that PAS could drive its effect through SST_2_ despite its higher binding affinity for SST_5_. However, the limited number of samples analyzed prevents the establishment of a conclusive correlation between SST differential expression and the anti-secretory effects of PAS, even though these results opened a new scenario because of their translational significance [126].

The role of SST_2_ as a mediator of a PAS anti-secretory effect on GH was confirmed in nine GH-secreting pituitary adenoma primary cultures, analyzing the effect of 10 nM OCT, PAS, and SRIF on GH release after 72 h of treatment [127]. It was reported that the three compounds inhibit GH secretion in a superimposable manner in almost all tested cultures (SRIF 6/6, OCT 7/9, and PAS 8/9). Similar to the study by Murray & Coll. [126], analysis of SST mRNA expression performed in seven of these tumor samples showed a predominant, although variable, expression of SST_2_ and SST_5_. However, apart from the previous study, a direct and significant correlation between SST_2_ mRNA expression and the anti-secretory activity of both OCT and PAS was demonstrated [127]. No correlation between the inhibitory effect of PAS on GH secretion and SST_5_ expression was found, despite the high binding affinity of this drug for this receptor subtype. This finding clearly suggest that the anti-secretory effect of PAS on GH-secreting adenoma cell cultures could be achieved via SST_2_. 

Subsequently, the effect of OCT and PAS on GH secretion was tested in a large number of GH-secreting pituitary adenomas (*n* = 32) [128]. SST_2_ and SST_5_ (with the latter more expressed than SST_2_) were the prevalent subtype expressed in GH-secreting pituitary adenoma cells, and the treatment of primary cultures with 100 nM OCT and PAS at different time-points (4 and 24 h) demonstrated superimposable effects of the two drugs in reducing GH secretion. Similar to GH reduction, a comparable effect of both compounds in lowering [Ca^++^]i, which is a pivotal second messenger involved in the regulation of hormone secretion, was reported, despite the number of OCT responsive samples (15/21) being slightly higher than samples responsive to PAS (11/21).

In another study, comparing 33 primary cultures derived from human somatotroph pituitary adenomas [129], GH levels after treatment with OCT and PAS were measured in correlation with the SST expression profile of each adenoma. In detail, OCT and PAS (10 nM for 72 h) demonstrated an overall comparable efficacy in GH reduction (36.8% vs. 37.1%, respectively). However, the analysis of a large number of samples allowed the authors to subdivide the adenomas in subgroups depending on their prevalent response to OCT or PAS, respectively. The PAS+ group, comprising six adenoma cultures (18%), which showed a better response to PAS, and OCT+ group, in which five samples (15%) showed a better response to OCT. SST_2_ and SST_5_ mRNA expression levels were correlated to the drug-dependent inhibition of GH release demonstrating, as expected, a significant positive correlation between SST_2_ expression and the anti-secretory activity of OCT. However, a slight, although not significant, trend of correlation was also found between SST_2_ expression and PAS-induced inhibition of GH secretion. Conversely, no correlation was found between SST_5_ expression and the anti-secretory effect of both drugs. The expression of SST_2_ as well as SST_2_/SST_5_ ratio, were significantly lower in PAS+ adenoma group, as compared to the other samples. However, among this group, higher amounts of SST_2_ mRNA were detected in the samples displaying ≥ 50% of GH inhibition, compared to samples with a lower response. Furthermore, a strong and direct correlation between the percentage of GH decrease induced by PAS and OCT was found by using a pairwise comparison of the different 33 adenoma cell cultures (*r* = 0.829, *p* < 0.0001), which strengthens the observation of a prevalent involvement of SST2 in mediating the biological action of both OCT and PAS [129].

All these studies demonstrated an overall comparable efficacy of the two drugs in reducing GH levels in somatotropinomas in vitro. Moreover, based on direct evidence of a positive correlation between SST_2_ mRNA expression and PAS efficacy [127,129], they highlighted a prevalent involvement of SST_2_ in the clinical effects, of both OCT and PAS.

Based on these results, several clinical trials aimed to evaluate the efficacy of PAS in acromegaly patients both medically naïve or non-responding to first generation SRLs (OCT and LAN), used as first-line medical treatment. A prospective, randomized, double-blind study compared OCT and PAS activity in 358 patients from 27 different countries [130], by enrolling medically naive acromegaly patients with GH >5 μg/L or GH nadir ≥1 μg/L after an oral glucose tolerance test (OGTT), and IGF-1 above the upper normal limit. Patients were treated with PAS LAR 40 mg/28 days (*n* = 176) or OCT LAR 20 mg/28 days (*n* = 182) for 12 months, which allows the possibility to increase the dose of both compounds (PAS LAR 60 mg or OCT LAR 30 mg) after three and/or seven months, if GH values were ≥2.5 μg/L and/or IGF-1 remain above the upper limit of normality. The main endpoint was the achievement of biochemical control (GH <2.5 μg/L and normal IGF-1) after the 12^th^ month. Results demonstrated that the percentage of patients who achieved biochemical control was higher in the PAS LAR treated group than in the OCT LAR group (31.3% vs. 19.2%, *p* = 0.007, 35.8% vs. 20.9%, when including patients with IGF-1 below the lower normal limit). In more detail, 38.6% and 48.3% of patients in the PAS LAR group achieved normal IGF-1 and GH <2.5 μg compared to 23.6% and 51.6%, respectively, in the OCT LAR group (*p* = 0.002). Patients who did not achieve biochemical control (31.0% of PAS LAR group and 22.2% of OCT LAR group) did not receive the recommended dose increase. The main adverse event was hyperglycemia, which shows a higher percentage of incidence in the PAS LAR group compared to the OCT LAR group (57.3% vs. 21.7%).

These results represent the first clear evidence of a higher efficacy of PAS LAR over OCT LAR, which allows PAS LAR to be considered a viable new treatment option for acromegaly [130].

In the same year, a randomized, clinical phase 3 trial comparing the effect of PAS with continued treatment with OCT or LAN in patients with inadequately controlled acromegaly (PAOLA study) was published [131]. Acromegalic patients, who were previously treated with 30 mg long-acting OCT or 120 mg LAN Autogel for ≥6 months as monotherapy, who did not normalize GH and IGF-1 levels (5-point, 2 h of mean growth hormone concentration >2.5 μg/L, and IGF-1 concentration >1.3 times the upper normal limit) were enrolled, and stratified according to the previous treatment (OCT or LAN) and GH concentrations at screening (2.5−10 μg/L and >10 μg/L). The patients were randomly assigned to PAS 40 mg (*n* = 65), PAS 60 mg (*n* = 65), or active control (*n* = 68) groups. After 24 weeks of treatment, 10 patients (15%) in the PAS 40 mg group and 13 patients (20%) in the PAS 60 mg group achieved biochemical control, which was not obtained in all the patients in the active control group. The most common adverse events were hyperglycemia (*n* = 21 [33%] for treatment with 40 mg PAS, *n* = 19 [31%] with 60 mg PAS, and *n* = 9 [14%] with active control) and diarrhea (*n* = 10 [16%], *n* = 12 [19%], and *n* = 3 [5%]). Most were grade 1 or 2 in severity. Serious adverse events were reported in six (10%) patients in the PAS 40 mg group, two (3%) in the PAS 60 mg group, and three (5%) in the active control group [131].

### 3.2. Cushing’s Disease

The presence of ACTH-secreting pituitary adenoma in the anterior lobe of the pituitary gland is the primary cause of Cushing’s disease (CD). In CD, the physiological negative feedback exerted by adrenal steroids is disrupted, which contributes to the autonomous ACTH hypersecretion from the adenoma. The resulting state of hypercortisolism is associated with peculiar clinical signs and symptoms and is correlated with a significant increase in patient morbidity and mortality [132].

When facing the challenge of the medical treatment of CD, it is well recognized that the ideal therapy should target the primary cause of the disease resulting in the control of hormone hypersecretion and reduction of the adenoma mass. This result still represents a “chimera” for clinicians and researchers, despite all the efforts and the different compounds developed and tested, especially in recent years [132].

Based on the well-recognized expression of SSTs on ACTH-secreting pituitary adenomas, first-generation SRLs, OCT, and LAN, were initially tested for the treatment of CD [133]. However, a lack of efficacy in suppressing both basal and CRH-stimulated ACTH secretion in patients was reported [133,134], likely because SST_2_, which is the main target of OCT and LAN, is significantly down-regulated by high levels of glucocorticoids occurring during active CD [135]. However, since SST_5_ is the most predominantly expressed SST subtype in human ACTH-secreting pituitary adenomas and in other CD cell models such as murine AtT20 cell line [136]. Its expression is not influenced by exposure to high glucocorticoid levels [137,138]. PAS has been identified as the main candidate in the medical treatment of CD. Several preclinical studies compared the effects of PAS and OCT in CD models before and after its approval in clinical practice.

An in vitro “head-to head” study evaluated the effect of PAS and OCT on ACTH release by six primary cultures of human corticotroph cells, as well as their effect on AtT20 cells [105]. The SST expression profile demonstrated a predominant expression of SST_5_ mRNA (> SST_2_) in all CD samples analyzed. As expected, treatment with 10 nM PAS for 27 h was more effective than OCT in the inhibition of basal ACTH secretion. PAS significantly decreased ACTH secretion in three out of five primary cultures (30% to 40%), while OCT inhibited ACTH release in only one out of five cultures (28% suppression). Similar results were obtained in AtT20 cells, in which only PAS, but not OCT, was able to inhibit with high potency (IC_50_ 0.2 nM) basal ACTH secretion [105]. Another detailed study used AtT20 cells to define the role of SST_2_ and SST_5_ in PAS and OCT-mediated inhibition of ACTH release, which reproduces the in vivo condition of glucocorticoids exposure observed during CD [137]. First, to better understand the role of SST_5_ in the inhibition of CRH-induced ACTH release, an experimental SST_5_-selective agonist (BIM-23268) was tested in addition to PAS, SRIF, and OCT, using culture conditions in the absence (basal condition) or presence of high levels of glucocorticoids. In basal conditions, PAS demonstrated a significantly higher potency (IC_50_ 0.06 nM) than OCT (IC_50_ 0.2 nM) in reducing ACTH secretion. Pre-treatment of AtT20 cells with 10 nM dexamethasone (DEX) led to a 20-fold decrease of OCT-induced inhibition of ACTH secretion (IC_50_ from 0.2 to 4.3 nM) while it did not affect a PAS anti-secretory effect.

The analysis of SST mRNA profile after DEX exposure for 48 h demonstrated a down-regulation of SST_2_ (45%), while SST_5_ expression was not affected. This supports, at a molecular level, the persistent effects of PAS in these experimental conditions, as compared to OCT [137]. In this context, the SST_5_ preferential compound BIM-23268 was significantly more effective in reducing CRH-induced ACTH release after DEX pretreatment than in basal conditions (60% vs. 15%). This finding could be related to possible modifications of the interactions among SSTs, such as SST_2_/SST_5_ crosstalk, induced by high levels of glucocorticoids, as well as to a possible effect of DEX on the intracellular machinery, which leads to an enhanced activation of the anti-secretory pathway [114,133,136].

In 2009, Ben-Shlomo & Coll. reported the effects of PAS, SRIF, and OCT, alone or in combination with selective receptor antagonists, on cAMP accumulation and intracellular Ca^++^ oscillation [139]. Authors used wild-type (WT) and transfected AtT20 cells, which overexpressed human SST_2_ or SST_5_ [139]. In WT cells, a significantly higher inhibition of cAMP accumulation was induced by PAS, as compared to both SRIF and OCT (IC_50_: 55 pM, 370 pM, and 470 pM, for PAS, SRIF, and OCT, respectively), as well as a more effective suppression Ca^++^ oscillation. However, different results were obtained in SST_2_ transfected cells, in which PAS was less effective in inducing cAMP reduction when compared to WT cells. As expected, co-treatment with the SST_2_-selective antagonist BIM-23454 did not affect PAS-induced cAMP reduction, which supports the evidence of a negligible involvement of this receptor subtype in PAS-mediated activity [139]. In SST_5_ transfected cells, the PAS effect was not enhanced by the high expression of the receptor, likely because, in the WT-setting, IC_50_ was already in the picomolar range. However, it induced a sustained efficacy on cAMP reduction, which suggests that SST_5_ can play a role in preventing cells from desensitization of different SSTs.

More recently, van der Pas & Coll. directly compared the efficacy of PAS and OCT on ACTH secretion in primary cultures of corticotroph pituitary adenomas from patients grouped according different levels of urinary free cortisol (UFC) measured before adrenalectomy [135]. In detail, they evaluated the effect of OCT and PAS on four primary cultures from patients with normal UFC levels before surgery, which demonstrates a significantly higher effect of PAS compared to OCT in inhibiting ACTH release in three out of four cell cultures (overall mean percent reduction: 49% vs. 26%, respectively). Although almost all samples showed a prevalent expression of SST2 mRNA, at the protein level, SST5 was expressed more in two out of four samples. Then, authors evaluated the effect of PAS and OCT on ACTH secretion in cells from a patient nearly reaching normal UFC before surgery (UFC 1.06 × ULN), which demonstrates that PAS was able to reduce ACTH levels at lower concentrations (IC_50_: 0.2 nM) than OCT, supporting previous reports [105]. Conversely, OCT significantly decreased ACTH secretion only at relatively high concentrations (IC_50_ 39 nM), which is in line with the results obtained by Ibáñez-Costa & Coll. [128].

These results strengthen the hypothesis of a major role of SST_5_ in driving the anti-secretory activity of PAS in ACTH-secreting pituitary adenomas when compared to OCT, even in the presence of higher expression of SST_2_ than SST_5_ mRNA in tumor cells [135].

On the other hand, different results were obtained by Ibáñez-Costa & Coll. who tested OCT and PAS on ACTH release and [Ca^++^]i kinetics in primary cultures of human corticotroph pituitary adenomas [128]. Treatments with 100nM OCT (three tumor samples) or PAS (four tumor samples) caused a significant ACTH reduction in two out of three tumors in the presence of high OCT concentrations, while only a slight ACTH reduction in two out of four samples was observed with PAS, despite a prevalent expression of SST_5_ mRNA in all samples as compared to SST_2_ mRNA. The effect of OCT and PAS on [Ca^++^]i was evaluated in 10 and nine primary cultures, respectively. Although both drugs prevented an [Ca^++^]i increase in about 50% of primary cultures (4/10 and 5/9, respectively), OCT was significantly more effective than PAS, as demonstrated by the proportion of responsive cells induced by the two compounds (56.1% OCT vs. 12.9% PAS) [128]. A possible explanation of these discordant results could be related to the supra-physiological concentrations of OCT used in this study (10-fold higher to the estimated therapeutic dose), which could have partially activated SST_5_, over SST_2_, despite the lower affinity of OCT for the latter receptor.

Moreover, most of the primary cultures were obtained from CD patients who underwent medical treatment before surgery and this condition could have caused an increase of SST2 mRNA levels, which leads to an SST_2_-related responsiveness [128]. However, the main pitfall concerning this study is related to the methodological setting of the experiments because these authors did not perform a head-to head comparison between OCT and PAS in the same cell culture, as in previous studies [105]. This experimental condition needs to be taken into account because the well-recognized inter-tumor heterogeneity of corticotroph pituitary adenomas, which could modify the responsiveness to medical treatments [140,141]. However, it is important to remark that all these data from preclinical studies were decisive for the approval of PAS as medical treatment for CD by both EMA and FDA.

In 2009, a multicenter Phase II clinical trial reported that PAS treatment decreased UFC levels and ACTH release in 76% of patients during a treatment period of 15 days [142]. The first phase 3 trial assessing PAS LAR in patients with CD was published in 2018 [143]. In this study, 150 patients with persistent, recurrent, or de-novo (non-surgical candidates) CD were enrolled: 74 patients (49%) were treated with 10 mg i.m. PAS LAR every four weeks for 12 months and 76 patients (51%) with 30 mg. The primary efficacy endpoint (mUFC normalization) was met by 31 out of 74 patients (41.9% [95% CI 30.5−53.9]) in the 10 mg group and 31 out of 76 patients (40.8% [29.7−52.7]) in the 30 mg group. Authors concluded that long-acting intramuscular PAS LAR displays a similar efficacy than the twice-daily subcutaneous injections, with positive responses in about 40% of patients with CD, which provides a convenient monthly administration schedule.

Lastly, a very recent study by Pivonello & Coll. evaluated the efficacy and safety of PAS treatment, according to the real-world evidence [144]. This study, performed on 23 CD patients, demonstrated that, in the real-life clinical practice, a six-month treatment with PAS normalizes, or nearly normalizes, UFC in about 68% of patients with a very mild to moderate disease. In line with the previously described clinical studies, the main adverse event was the induction or worsening of glucose imbalance, which confirms the usefulness of this treatment in patients with mild disease, without uncontrolled diabetes.

### 3.3. Other Pituitary Tumors

Because other pituitary adenomas, such as prolactinomas, TSH-secreting and FSH-secreting adenomas and non-functioning pituitary adenomas (NFPAs) express different SST subtypes besides SST_2_ and SST_5_, and/or other GPCRs, such as D2R. Additional medical treatments are used in these tumors (e.g., D2R-preferential drugs, such as cabergoline, in prolactinomas). Moreover, pituitary tumors such as TSH-secreting and FSH-secreting adenomas are very rare. Therefore, only a few studies evaluated the effects of OCT and PAS in these tumors.

Ibáñez-Costa & Coll., in the previously mentioned study [128], compared the effect of OCT and PAS in four primary cultures of prolactinomas and in 28 NFPAs measuring [Ca^++^]i and cell viability. 

Primary cultures were established from prolactinomas, which displayed in vivo resistance to cabergoline treatment. Analysis of the SST profile demonstrated high levels of SST_1_ associated with a low expression of the other SSTs. Treatment of cells with OCT and PAS showed a poor effect on [Ca^++^]i of both SRLs: OCT decreased [Ca^++^]i in two out of four cultures, but only in 7.0% of the cells, while PAS failed to induce appreciable effects. Both SRLs decreased cell viability in one-third of tumors after 48 to 72 h.

As far as NFPAs, the SST expression profile demonstrated a high expression of SST_3_, followed by SST_2_, SST_5_, SST_1_, and SST_4_, which suggests a possible prevalent effect of PAS compared to OCT. Despite SST expression profile, both compounds showed a poor effect on [Ca^++^]i kinetics inhibition even though OCT was slightly more effective than PAS in an isolated subset of cells [128]. Discordant results were obtained in the evaluation of cell viability. NFPA primary cultures differently responded to OCT and PAS treatment showing a slight and no significant decrease in cell viability after treatment with OCT (5/16 cell cultures after 24 h, 2/8 after 48 h, and 2/7 after 72 h), as well as with PAS (4/15 cell cultures after 24 h, 1/7 after 48 h, and 1/4 after 72 h). Conversely, a moderate significant increase in cell viability was induced by treatment with OCT when compared to untreated cells in 11/16 cell cultures after 24 h, in 5/8 cell cultures after 48 h, and in 2/6 cell cultures after 72 h. Similarly, to OCT, PAS increased cell viability in in 11/15 NFPA cell cultures after 24 h, in 6/7 after 48 h, and in 3/4 after 72 h.

In three TSH-secreting pituitary adenoma primary cultures, the SST_2_/SST_5_ expression ratio was predictive of the hormonal and cell viability effects of OCT, likely due to the prevention of tachyphylaxis. However, the association of D2R agonists to SRLs could prevent the pharmacological escape [145].

To sum up, based on preclinical evidence, we can reasonably state that OCT and PAS can alternatively act by activating SST_2_ and/or SST_5_ depending on the specific tumor cell type. Indeed, as expected, OCT is mainly effective in SST_2_-expressing GH-secreting cells as well PAS in SST_5_-expressing tumor corticotroph cells. However, a number of results showed that PAS is also able to inhibit GH secretion in somatotropinomas by activating SST_2_, despite its prevalent affinity for SST_5_. Similarly, high doses of OCT can act through the activation of SST_5_ in some corticotropinomas, which highly expresses this receptor subtype.

**Figure 3:**
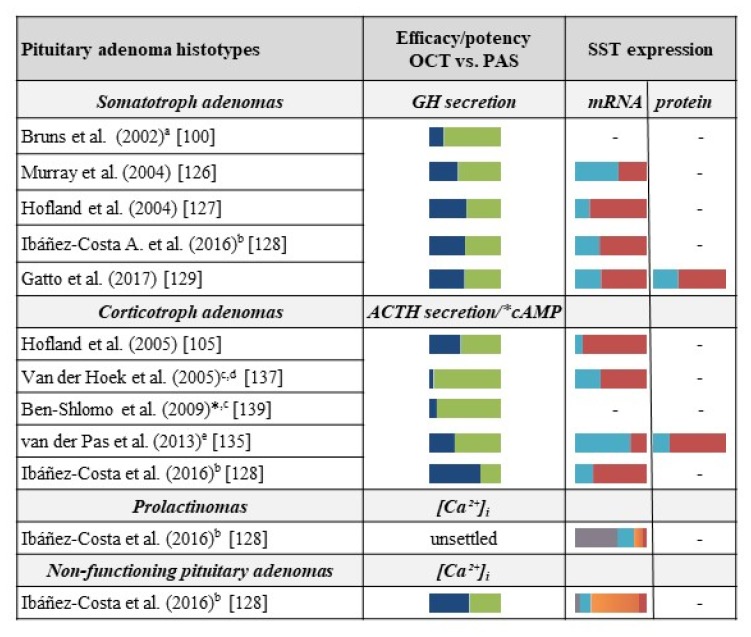
Differential effects of OCT and PAS on pituitary adenomas, in relationship with the SST profile. In the left column, blue = OCT and green = PAS. In the right column, light blue = SST2, red = SST5, grey = SST1, and orange = SST3. cAMP, intracellular cAMP level. [Ca²⁺]i, intracellularcalciumconcentration. ^a^ GH release by primary cultures of rat anterior pituitary cells, after 6 h of treatment. ^b^ Data extracted from graphs presented in the related article. ^c^ Study carried out using the AtT20 cell line. ^d^ Effect of OCT and PAS on CRH-induced ACTH release after pre-treatment with DEX and mRNA expression after 24−48 h of DEX. ^e^ Primary cultures from patients with normalized urinary-free cortisol. * Studies in which changes in cAMP accumulation in response to OCT or PAS was analyzed.

Figure 3 summarizes all the reported data as far as the effects of OCT and PAS on pituitary adenomas, in relationship with the SST profile evidenced in the respective study.

## 4. Comparison between First- and Second−Generation Somatostatin Receptor Ligands in Neuroendocrine Neoplasms

Neuroendocrine neoplasms (NENs) comprise clinically and biologically heterogeneous tumors, originating from neuroendocrine cells disseminated in different organs (e.g., pancreas, stomach, lung, and colon) [146]. The most common NENs occur in the gastrointestinal tract or pancreas (GEP-NEN) and bronchopulmonary system [147], with an incidence increasing over the years, likely due to more efficient diagnostic tools [148]. Regardless of the anatomic origin, NENs include neuroendocrine carcinomas (NECs), characterized by poor differentiation and high-proliferation rate, and neuroendocrine tumors (NETs), well-differentiated and low-proliferating lesions, whose metastatic potential depends on tumor type, localization, and grade. By means of Ki-67 index and mitotic count, WHO 2017 classification grades NENs as: low-grade NET G1 (Ki-67 < 3%, mitotic index <2/10 hpf), NET G2 (Ki67 3-20% or mitotic index 2-20/10 hpf), while NECs are by definition G3 (Ki67 index >20% or mitotic index >20/10 hpf). G3 pancreatic NENs include both differentiated low proliferating NETs and undifferentiated aggressive small or large cell NECs [149]. These grading parameters reflect biologic aggressiveness of NENs and have prognostic and therapeutic relevance. NETs are also divided in two subgroups: (i) non-functioning tumors, which are the most common and do not secrete detectable levels of hormones with no or non-specific clinical symptoms [150], (ii) functioning tumors whose hormone hypersecretion leads to distinct signs of disease (flushing, diarrhea, hypoglycemia, hyperglycemia, peptic ulcers, etc.), and express biomarkers of neuroendocrine differentiation such as chromogranin A (CgA) and synaptophysin [151], as well as peptide hormones.

Furthermore, a specific staging system has been developed for GEP-NENs, which is the classic tumor/node/metastasis (TNM) classification and a clinical staging for mortality risk assessment based on the anatomical extent of NEN [152]. This is useful for decision-making regarding treatment.

The great majority of GEP-NENs, as well as approximately 50% of small intestine NETs and insulinomas, express multiple SST subtypes involved in hormone secretion and tumor cell growth. SST_2_ expression is predominant, followed by SST_5_, while SST_1_, SST_3_, and SST4 are scarcely detected, even if a great variability among NET types and localization was observed [153,154,155]. Moreover, molecular investigation revealed that pNETs also express a truncated splice variant of the SST5 (SST5TMD4) [156]. In GEP-NEN, SST_2_ expression is inversely correlated with grading and patient outcome, as well as the expression of the chemokine receptor CXCR4 [153,157]. Similarly to SSTs, CXCR4 is often present in highly proliferative and advanced tumors of various origin, and the chemokine-receptor system CXCL12-CXCR4 largely influences neuroendocrine regulation and functions [158,159]. It may represent an additional diagnostic and therapeutic target in NENs [160].

### 4.1. Somatostatin Receptor Ligands in Preclinical Models of Neuroendocrine Neoplasms

The small incidence and large heterogeneity of NENs, along with inadequate availability of disease experimental models, often prevents a satisfactory preclinical and translational research. Nevertheless, knowledge of molecular alterations associated with NEN pathogenesis and progression (e.g., activation of mTOR signaling and tyrosine kinase activity of PDGFR and VEGFR) has improved over recent years, by favoring the identification of targeted drugs such as everolimus [161] and sunitinib [162], which currently are complementary to SRLs as medical management of NET clinical symptoms [19].

The paucity of NEN-derived cell lines (the two most used are the human pancreatic carcinoid cell, BON-1, and the human pancreatic islet cell carcinoma cell line, QGP-1, which show mutations in accordance with those detected in sporadic pNET patients) [163], and the technical issues in the establishment of patient-derived primary cultures and xenografts, makes preclinical research difficult to adequately recapitulate in vitro NET disease. Moreover, the usefulness of cell lines as a model for studying NEN responses to SRLs is debatable.

First, the in vitro activity of OCT was tested in BON-1 cells, which express SST_1_, SST_2_, and SST_5_: OCT inhibited cAMP accumulation, CgA secretion, and MAP kinase activity [164], Akt-phosphorylation and cell growth [36], and tumor growth [165]. On the contrary, other studies reported the lack of significant anti-proliferative effects in both BON-1 and QG-1 cells [166,167] neither in 2D cultures nor in 3D-spheroid models [168], as well as in mice bearing BON-1 xenografts [166]. The BON-1 cell line has been also proposed as a model mimicking the IGF system on GEP-NETs and a model used to test the activation of IGF-related factors upon treatment with OCT and PAS [169].

OCT also failed to exert anti-proliferative activity in the most frequently used NET cell lines, which showed SST_2_ expression levels lower than observed in human NEN tissues [165]. Overall, the lack of SRL effects might be explained by: (i) low SST_2_ expression in BON-1 and other cell lines, (ii) limited similarity of GEP-NET cell lines with a tumor phenotype, and (iii) cancer-associated mutations occurred in cell cultures [170]. This implies a careful extrapolation of results, which encourages a shift to patient-derived cultures and xenografts.

The NET cell line QGP-1 was also used to analyze SST_2_ domains regulating receptor trafficking and signal transduction: the treatment with the SST_2_-specific analog BIM23120 caused SST_2_ desensitization and internalization, which possibly explains SRL resistance detected in vivo after prolonged treatment with SST_2_ preferential compounds [171].

A better understanding of SRL effects was obtained after isolation and characterization of primary NET cultures. Cell cultures from 15 human pNETs were shown to retain in vitro SST expression profile (predominantly SST_2_) and a secretory phenotype (CgA) of the original tumor, and were used to explore and compare the biological activity of OCT and PAS [117]. OCT reduced both cell viability and CgA release via SST2 activation, which, however, rapidly results in prolonged receptor internalization. On the contrary, PAS, which similarly exerts anti-proliferative and anti-secretory effects but does not phosphorylate SST_2_, induced a fast and transient receptor internalization/membrane trafficking cycle. Therefore, primary cultures explain clinical observations of tachyphylaxis induced by OCT in GEP–NET patients [117,172,173], together with modulation of downstream pathways [173]. The observation of a lower desensitization induced by PAS might imply that its activation of SST_5_ stabilizes SST_2_ at the cell membrane, which forms a heterodimer that sustains long-lasting efficacy of SRLs.

Human pNET primary cultures were also used to test the possible synergism as far as the anti-tumor and anti-secretory activity of SRLs and everolimus [174], which is a mTOR inhibitor approved for advanced pancreatic, gastrointestinal, and lung NETs [175], to elucidate possible mechanisms at the basis of disappointing efficacy and resistance occurrence observed in patients. Everolimus inhibited CgA production and cell viability, but co-treatment with SRLs neither improved its efficacy nor circumvented everolimus-dependent Akt activation [174]. Again, the primary culture model closely mimics the lack of synergistic/additive anti-tumor effects of SRLs in combination with everolimus, as observed for overall survival in patients. Mutations and alterations of the mTOR pathway in subsets of NETs [176,177] as well as preclinical efficacy of everolimus in NET cell lines [166,177,178,179,180] were extensively reported, and provided the rationale for mTOR inhibitor therapy. However, remarkable clinical benefits were limited and still debated [181]. mTOR inhibition leads to multiple compensatory pro-survival signals (e.g., activation ERK1/2 and PI3K/Akt pathways) [181], which could explain everolimus resistance as reported in primary NET cultures [174].

### 4.2. Somatostatin Receptor Ligands in Clinical Management of Neuroendocrine Neoplasms: Improvement of Symptoms and Anti-tumor Effects

Due to NENs heterogeneity, multimodal treatment strategies such as surgery, systemic pharmacological treatment, and radiotherapy are used to control clinical symptoms due to hypersecretion and tumor growth, and to delay disease progression [182]. Although surgery represents the first line treatment of NENs, a high percentage of tumors is not surgically resectable because of their metastatic condition at the time of initial diagnosis (mainly small intestine and pNETs). In this condition, the use of medical treatment represents the main tool to control tumor-related symptoms and to prolong patient survival.

First line systemic treatment of well differentiated, locally advanced or metastatic NETs consist of SRLs (OCT LAR and LAN Autogel). Alpha-interferon and targeted drugs, such as everolimus, sunitinib, and bevacizumab, an anti-VEGFA antibody, or other tyrosine kinase receptor targeting antibodies and small molecules [183], are additional treatment options. Chemotherapy for advanced poorly or well differentiated NETs with a high-tumor burden [184].

GEP-NET patients progressing or recurring after failure of initial therapies have limited treatment options. Therefore, peptide receptor radionuclide therapy (PRRT) with SRL-based ^177^ Lu-DOTATATE, ^90^ Y-DOTATATE, or ^90^ Y-DOTATOC could be further treatment options [185]. G1 and G2 NENs satisfactorily respond to PRRT, while the response in G3 NENs is still unclear. Therefore, guidelines for G3 NECs did not recommend radiopharmaceutical therapy [186,187].

OCT and LAN (both immediate-release and long-acting formulations) are the standard of care for functioning and non-functioning NETs [182,188] to control severe symptoms (diarrhea and flushing, carcinoid syndromes) associated with hypersecretion of serotonin, and pancreatic islet cell hormonal-secretory syndromes sustained by gastrin, glucagon, insulin, vasoactive intestinal peptide (VIP), and parathyroid hormone-related peptide [189].

First-generation SRLs have been widely demonstrated to control symptoms in patients with NETs [190,191,192]. Anti-tumor efficacy, as well as time to tumor progression, was prolonged from 6 to 14.3 months, which was observed with OCT LAR in the PROMID Phase 3 study analyzing metastatic midgut NETs [24], and significant increases in PFS was reported in LAN Autogel-treated patients entered in the placebo-controlled Phase 3 CLARINET trial [23]. These results encouraged FDA and EMA approvals for clinical use in GEP-NETs. Both OCT and LAN are highly effective in managing carcinoid symptoms [189], which shows an increase in patients’ PFS [27] and good tolerability [193].

The response to SRL treatment in terms of inhibition of secretion and tumor growth is extremely variable among NEN patients, likely due to both inter-tumor variability and differences in the development of resistance to therapy, besides pharmacological properties of SRLs (biased agonistic activity). After prolonged treatments with first generation SRLs, GEP-NET patients often exhibit inadequate control of symptoms due to hormonal overproduction at conventional doses. Therefore, resistance due to tachyphylaxis is a frequent clinical problem [194]. Several mechanisms underlying SRL resistance have been proposed including SST_2_ downregulation/internalization or stabilization/degradation [172,173], as previously discussed, and changes in SST signaling/functioning and upregulation of proliferative genes [195]. At least initially, higher doses successfully counteract the loss of susceptibility to SRLs, which allows an adequate disease control and maintains good tolerability. However, the outcome in terms of PFS and overall survival (OS) with the escalation-dose are still unclear [196].

Preclinical studies, showing the lower impact of PAS on SST_2_ phosphorylation, internalization, and recycling [104,117], suggested the potential of this agent to overcome tachyphylaxis and prolong the response. OCT LAR-refractory GEP-NET patients treated with PAS achieved a satisfactory relief of diarrhea and flushing (~30% of patients) and tolerability in a Phase II study [197]. Subsequently, a Phase 3 trial in advanced and symptomatic patients after SRL treatment exploring the efficacy of PAS did not show benefits when compared to high doses of OCT LAR [198].

PAS is not routinely used in the treatment of NETs since the evidence of efficacy of this drug in patients progressing after OCT or LAN treatments is still scanty and rather controversial [198,199], even in escalated-doses studies [200]. In addition, the frequent occurrence of hyperglycemia (~80% of patients) observed with this drug [201], likely due to the effect of SST_5_-mediated reduction of insulin production by pancreatic β-cells, makes its risk/benefit profile questionable. Further studies will help clarify the reliability of PAS, alone or in combination with other agents [199,202], in the management of NETs.

In summary, currently OCT LAR and LAN Autogel remain the cornerstone of systemic therapy for NETs, either alone, or in combination with other targeted therapies [203]. Advances in the knowledge of the SST pathways, which are molecular and microenvironmental mediators relevant for the pathogenesis of these tumors, will be crucial to discover novel therapeutic targets and improve anti-proliferative efficacy and safety of the drugs currently used in the management of NENs.

## 5. Conclusions and Future Perspectives

First-generation SRLs (OCT and LAN) primarily differ from the second-generation SRL (PAS), for their peculiar membrane receptor binding affinity. While OCT and LAN are mainly SST_2_-preferential ligands, PAS shows high binding affinity for multiple receptor subtypes (predominantly SST_5_, SST_2_, and SST_3_). However, a number of preclinical studies published in the last decade have demonstrated that the differences between first-generation and second-generation compounds go far beyond the mere SST binding affinity. In most reports, PAS showed a peculiar pattern of receptor phosphorylation, likely due to the activation of ligand-specific changes in receptor conformation. Therefore, PAS treatment results in a specific activation of intracellular pathways, as well as in a peculiar target-receptor internalization and trafficking compared to both OCT and native SRIF, mainly when evaluating SST_2_ (the SST mostly expressed in the majority of pituitary adenomas and NENs).

However, these observations mainly result from preclinical studies carried out on transfected cell models, which do not always represent reliable models for pituitary adenomas and NENs. Furthermore, evidence from data collected using tumor primary cultures suggests that the biological effects of a pan-ligand such as PAS may also vary depending on the specific tumor type. This hypothesis is supported by the evaluation of studies carried out on somatotroph and corticotroph adenomas. While in GH-secreting pituitary cells, PAS seems to act mainly via SST_2_. Its effect is predominantly exerted through SST_5_ in corticotroph cells. Of note, the biased-agonist effect observed after PAS binding to SST_2_ in transfected cell models has not been confirmed when using cells from somatotroph primary cultures, which endogenously express the receptors and represents a more reliable preclinical model of acromegaly. On the other hand, the higher potency and efficacy of PAS, in comparison to both OCT and LAN when targeting SST_5_ in transfected cell models is confirmed by preclinical evidence on human corticotroph adenoma cells (mainly expressing SST_5_). Although it was not formally demonstrated, these discrepancies observed in different cell types might also reflect the general receptor profile of the cells, which included the presence of receptors other than SSTs that can influence the SRL responses due to heterodimerization pathways. Further studies are warranted to address this issue.

In conclusion, data published so far demonstrate that, despite the initial aim to generate a drug with a universal binding profile for SSTs with biological characteristics similar to that of native SRIF, the second-generation SRL PAS shows cell and tissue specific properties that are not completely understood yet. In our opinion, future research studies should aim to go deeper and investigate the peculiar biological mechanisms activated by PAS (in comparison to first-generation SRLs) in preclinical models that are able to closely mimic the behavior of NEN in vivo. Although we are aware that this represents a huge challenge for the scientific community, some steps forward have been achieved in recent years (i.e., zebrafish xenografts, identification of pituitary adenoma cell hierarchy [204,205]), and new tools may help reach these goals).

## Figures and Tables

**Table 1:** Trafficking properties of transfected human and rat somatostatin receptor subtype 2 (SST_2_) after binding to endogenous somatostatin (SRIF), octreotide (OCT), and pasireotide (PAS).

SST_2_ Trafficking Properties	Ligand	Receptor Internalization (EC_50_, nM)	Maximal Internalization (% of total) ^a^	Receptor Recycling (min) ^b^
hSST_2_ *	SRIF	3.26	−	n.a.
	OCT	6.46	−	n.a.
	PAS	31.78	−	n.a.
rSST_2_ **	SRIF	0.4	82.8	42.3
	OCT	n.a.	n.a.	n.a.
	PAS	23.3	46.0	4.8

hSST_2_, human SST_2_. rSST_2_, rat SST_2_. EC_50_, half-maximal effective concentration. min, minutes. n.a. not assessed. In most preclinical studies, pasireotide (PAS) was named as SOM230.* Data about hSST_2_ are reproduced from Reference [102]. ** Data about rSST_2_ refers to Reference [107]. ^a^ Internalization of hSST2 is evaluated after 30 min of SRIF, OCT, and PAS treatment at 1 µM concentration, while internalization of rSST2 is assessed after 90 min of SRIF 100 nM and PAS 1 µM concentration. ^b^ rSST2 recycling was measured after 30 min of SRIF 100 nM and PAS 1 µM concentration.
